# The complete mitogenome of ‘Dongfang No.3’, an important *Saccharina* cultivar in China

**DOI:** 10.1080/23802359.2021.1899863

**Published:** 2021-03-19

**Authors:** Yu Xia, Zhenyi Guo, Xinyi Zhang, Jing Zhang

**Affiliations:** Shandong Academy of Sciences, Qilu University of Technology, Jinan, PR China

**Keywords:** Cultivar ‘Dongfang No.3’, mitogenome, phylogenetic relationship, *Saccharina*

## Abstract

In this work, the complete mitogenome of *Saccharina* cultivar ‘Dongfang No.3’ is reported. This mitogenome has a circular mapping organization with the length of 37,657 bp and contains 66 genes, including 35 protein-coding genes, three rRNAs, 25 tRNAs, and three open reading frames (*orf*s). The overall AT content is 64.73%, showing a higher AT content. The gene content and gene sequence are consistent with those reported varieties and cultivars of *Saccharina*. Chinese main *Saccharina* cultivars are analyzed by phylogenetic analysis. It indicates that ‘Dongfang No.3’ has a close relationship with *Saccharina japonica*, which strongly supports its genetic origin. The complete mitogenome analysis in this work would help in understanding the genetic background of Chinese *Saccharina* cultivars.

*Saccharina* (Laminariales, Phaeophyceae) is an economically important brown macroalgae (Kain 1979) and has made important contributions to Chinese mariculture. ‘Dongfang No. 3’, as hybrid of *Saccharina japonica* and *Saccharina longissima*, is resistant to high temperature and strong light (Cong 2009). It has been approved by the China Aquaculture Superior Species and Original Species Approval Committee (GS-02-002-2007). However, its information on genomics is limited. Now, we characterized the complete mitogenome of *Saccharina* cultivar ‘Dongfang No. 3’ and performed phylogenetic analysis to provide new genomic data for genetic research on this excellent *Saccharina* cultivar.

The ‘Dongfang No. 3’ specimen (specimen number: 201008370) was collected from Yangma Island, Yantai City, Shandong Province, China (37°47′N, 121°62′E), and stored in the Culture Collection of Seaweed at the Ocean University of China for DNA isolation. The homologous PCR amplification method was described by Zhang et al. (2011). PCR primers are shown in the Supplementary material. Sequencing reactions were conducted by the ABI 3730 XL automated sequencer (Applied Biosystems, Foster City, CA). All obtained sequences were edited and assembled using the DNAStar (DNASTAR, Inc., Madison, WI). The protein-coding genes, *rRNA* genes and *tRNA* genes were annotated based on the reference sequences of *Saccharina japonica* (GenBank accession number AP011493) using Geneious R10 (Biomatters Ltd., Auckland, New Zealand; available from http://www.geneious.com/).

The mitogenome of ‘Dongfang No.3’ contains a circular molecule with 37,657 bp in length (GenBank accession number MG712777, https://www.ncbi.nlm.nih.gov/nuccore/MG712777.1/). Its total AT content is 64.73%, with a nucleotide composition of 28.40% A (10,696), 14.69% C (5533), 20.58% G (7748), and 36.33% T (13,680). The cumulative GC-skew and AT-skew analysis reflect the slight bias of G and T on the H-strand. The mitogenome encodes three rRNAs (23S, 16S, and 5S), 25 tRNAs, 35 protein-coding genes, and three *orf*s (*orf*41, *orf*130, and *orf*377). The length of the protein-coding region is 29,007 bp, accounting for 77.03% of the entire mitogenome. With the exception of *rpl*2, *rpl*16, *rps*3, *rps*19, *tat*C, and *orf*130, sixty genes are encoded on H-strand. The universal genetic codes are used and all start codons are ATG. Of the 35 protein-coding genes and three *orf*s, 26 (68.42%) terminated with TAA codon, higher than that for TAG (8, 21.05%) and TGA (4, 10.53%). The conserved gene cluster (*rps*8-*rpl*6-*rps*2-*rps*4) in the reported *Saccharina* mitogenomes is also found here. Furthermore, all *tRNA* genes are provided with standard clover-leaf secondary structures. Gene content and gene order show a high level of conservation with those reported *Saccharina* mitogenomes (Yotsukura et al. 2010; Zhang et al. 2011; Zhang et al. 2013; Zhang et al. 2017; Zhang et al. 2018).

Compared with the mitogenome of *S. japonica*, ‘Dongfang No. 3’ has 35 nucleotide substitutions. Among them, 16 substitutions are found in the protein-coding region. Thirteen of the above substitutions caused the changes of amino acid sequences and none of them changes the types of proteins.

Phylogenetic relationship of 19 *Saccharina* and *Laminaria* algae was conducted using amino acid sequences from 35 shared protein-coding genes by maximum likelihood (ML) and Bayesian inference (BI) methods (Figure 1). *Ectocarpus siliculosus* served as an out-group. Each amino acid sequence was aligned individually using MEGA7 and then the concatenated alignment was generated by BioEdit. The concatenated alignments with conserved regions were produced by the Gblocks server (http://molevol.cmima.csic.es/castresana/Gblocks_server.html) (Castresana 2000). ML analysis was performed using RAxML (Stamatakis 2006) with 1000 replications under the CpREV + G + I + F model. BI was performed based on MrBayes version 3.1.2 (Huelsenbeck and Ronquist 2001) using CpREV model. The phylogenetic analysis was performed using two independent runs with four Markov Chains, which ran for 1,000,000 generations until the average standard deviation of split frequencies was below 0.01. Output trees were sampled every 100 generations. FigTree version 1.4.3 (http://tree.bio.ed.ac.uk/) was used to display the phylogenetic tree (Rambaut 2016). Phylogenetic analysis shows that all algae are divided into two branches: *Saccharina* and *Laminaria*. Including ‘Dongfang No.3’, all Chinese cultivars belong to the *Saccharina* lineage. ‘Dongfang No.3’ has a close relationship with *S. japonica* supporting its genetic origin. The phylogenetic tree in this work also provides more information for the current understanding of genetic relationships among Chinese *Saccharina* cultivars.

**Figure 1.**
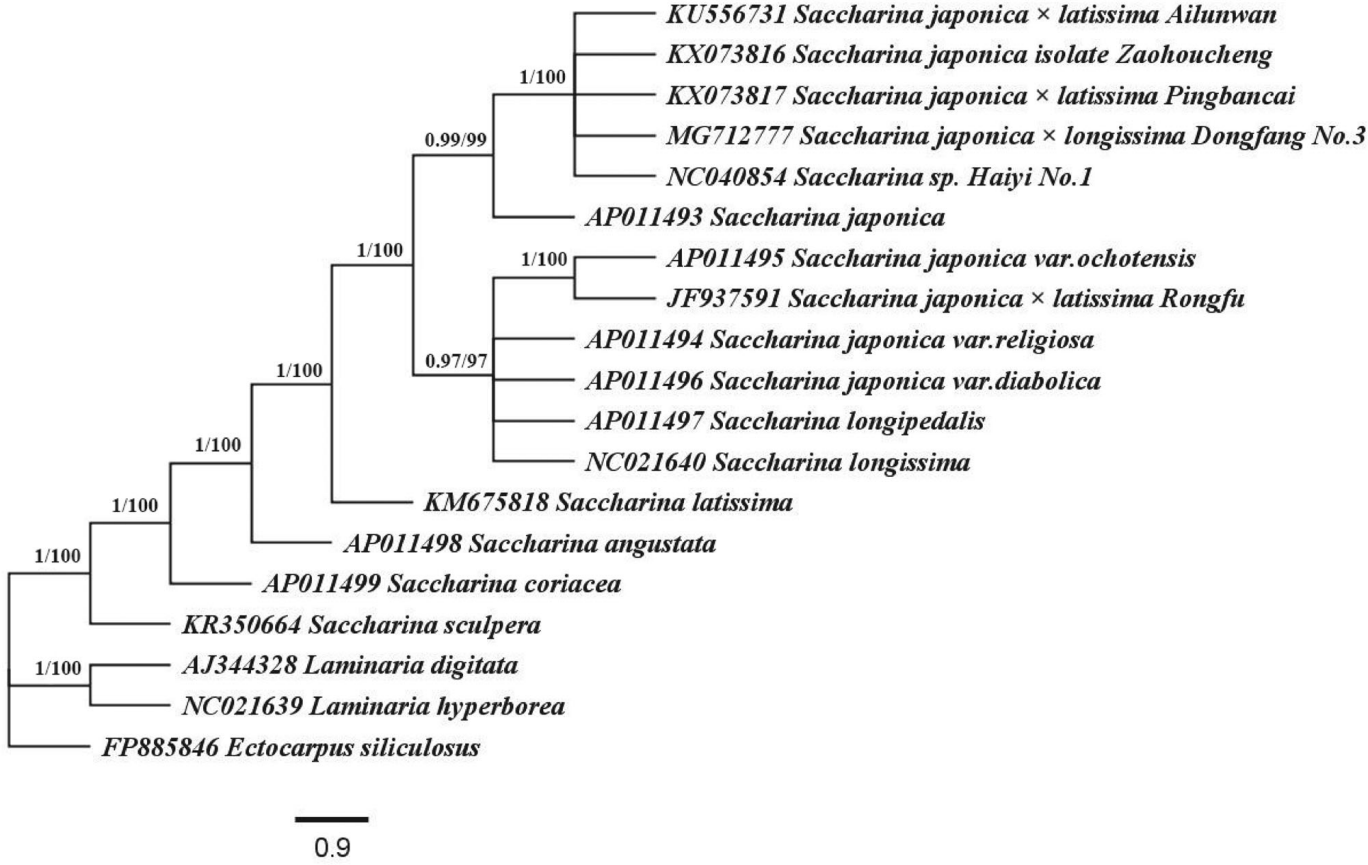
Phylogenetic tree constructed based on 35 shared protein coding genes from 19 mitochondrial genomes of *Saccharina* and *Laminaria* algae using BI and ML methods.

## Data Availability

The genome sequence data that support the findings of this study are openly available in GenBank of NCBI at https://www.ncbi.nlm.nih.gov/nuccore/MG712777.1/under the accession number MG712777.1.
